# Machine Learning-Assisted DFT Screening of Nitrogen-Doped Graphene Diatomic Catalysts for Nitrogen Reduction Reaction

**DOI:** 10.3390/molecules30204131

**Published:** 2025-10-20

**Authors:** Xiulin Wang, Suofu Nie, Huichao Yao, Sida Wu, Yanze Li, Junli Feng, Yiyan Sui, Yuqing Zhang, Xinwei Wang, Xiuxia Zhang

**Affiliations:** 1 CNOOC Key Laboratory of Liquefied Natural Gas and Low-Carbon Technology, Beijing 100028, China; 2 CNOOC Gas & Power Group, Research & Development Center, Beijing 100028, China; 3 Department of Energy and Power Engineering, College of New Energy, China University of Petroleum (East China), Qingdao 266580, China

**Keywords:** dual-atom catalysts, machine learning screening, nitrogen-doped graphene, nitrogen reduction reaction, density functional theory calculation

## Abstract

This research seeks to investigate extremely efficient catalysts for the nitrogen reduction process (NRR), utilizing machine learning (ML)-aided density functional theory (DFT) computations. Specifically, we investigate dual transition metal atoms anchored on hexagonal nitrogen-doped graphene (TM_1_-TM_2_@N_6_G) as prospective high-activity catalysts for the NRR. The findings indicate that the synergistic effect of dual transition metal atoms in the TM_1_-TM_2_@N_6_G catalyst overcomes the intrinsic constraints of the linear relationship among intermediates, facilitating the activation and adsorption of N_2_, thereby exhibiting significant potential for ammonia synthesis through N_2_ reduction. Particularly, four catalysts screened by ML and DFT exhibit good stability and excellent selectivity and activation towards N_2_. Among them, the catalysts Ti-Cr@N_6_G, Ti-Mo@N_6_G, and Ti-Pd@N_6_G possess two reaction pathways with minimum reaction energies of 0.55 eV, 0.50 eV, and 0.40 eV, respectively. Remarkably, Ti-Co@N_6_G, which features a single reaction pathway, exhibits a reaction energy lower than 0.05 eV, allowing the NRR to proceed spontaneously. It is noteworthy that incorporating ML into DFT calculations facilitates the rapid screening of all transition metal combinations, significantly accelerating the research on catalytic performance and optimizing the selection of catalysts.

## 1. Introduction

Ammonia, as an important inorganic chemical product and raw material, plays a pivotal role in the production of nitrogen fertilizers and nylon and holds potential for hydrogen storage [1] and zero-carbon energy sources [2]. However, the primary method for ammonia synthesis in industry is the Haber–Bosch process (N_2_(g) + 3H_2_(g) → 2NH_3_(g)), which activates inert N_2_ (with a molecular bond energy of 945 kJ/mol) and converts it to NH_3_ with the aid of iron or ruthenium-based catalysts under high-temperature and high-pressure conditions (350–550 °C, 150–350 atm) [3,4]. This process is plagued by issues such as high energy consumption, significant CO_2_ emissions, and low conversion rates, failing to meet the demands for environmental protection and green catalysis in the context of the “dual carbon” strategy. Furthermore, the harsh production conditions result in the extensive use of fossil fuels, which also adversely affects the environment [5]. Although the nitrogen source in NRR technology is readily available, the hydrogenation reaction is difficult to achieve due to the chemical inertness of the N≡N bond, significantly reducing the catalytic reaction rate and imposing stringent requirements on reaction conditions. Therefore, there is an urgent need to develop a highly active novel catalyst that can achieve ammonia synthesis under low-temperature and low-pressure conditions, thereby reducing costs and technical difficulties.

In the past decade, the emergence of atomic-scale catalysts has injected new vitality into the field of catalysis. These special supported metal catalysts (SACs) boast high atom utilization, high catalytic activity, controllable selectivity, and good stability, garnering increasing attention [6,7,8,9,10]. Numerous experiments and studies have demonstrated that isolated single atoms, particularly transition metal atoms embedded or anchored on suitable substrates, can effectively catalyze NRR [11,12,13,14]. However, due to the existence of isolated active centers, it is difficult to carry out multi-atom synergistic catalysis [15]. To further enhance atom utilization and consider the impact of different atom combinations on the catalytic process, researchers have focused on introducing new metal atoms into SACs to create dual-atom catalysts (DACs). It has been found that these catalysts not only improve the loading rate of metal atoms and functional active sites but also achieve efficient atom utilization. The two metal atoms can cooperate to effectively tune their d-band centers, enhancing the interaction between reactants or intermediates and catalytically active sites [16,17,18]. However, the diversity of combinations for such dimetallic active sites significantly increases the workload in the search for highly active NRR metal diatomic catalysts.

In recent years, with the rapid advancement of machine learning methods, the speed and efficiency of theoretical catalyst screening are expected to see significant improvements. Traditional empirical models are often constrained by limited chemical intuition and trial-and-error approaches, leading to inefficient catalyst development and lengthy cycles [19]. In contrast, machine learning technology can accelerate the discovery and optimization of new catalytic materials by swiftly identifying potential high-activity catalyst structures and guiding experimental design through big data analysis and model predictions. This innovation not only helps reduce costs but also drives progress in catalytic science and industry for key reactions such as ammonia synthesis, providing crucial support for sustainable development. Consequently, a growing number of studies have recently explored the use of machine learning (ML) methods for catalyst discovery and design [20,21,22,23,24]. Machine learning typically relies on data-driven models that utilize regression or classification algorithms for training on existing datasets. Once trained, these models can predict the target variable, or desired outcome, based on the key features of the input. Therefore, machine learning serves as a powerful tool for researchers to quickly screen potential catalyst candidates within the vast chemical space. By analyzing extensive chemical data and physical properties, these algorithms can identify catalyst structures with high activity and selectivity, thereby expediting the new material development process. This approach not only enhances the efficiency of catalyst design but also offers valuable guidance and optimization directions for laboratory research [25,26]. Compared to traditional theoretical calculations like density functional theory (DFT), ML-assisted DFT can significantly reduce computational costs, better comprehend the potential relationship between material structure and catalytic performance, and effectively identify intrinsic catalyst descriptors [16,17,18,26,27,28,29,30,31,32,33]. Therefore, when combined with ML-assisted DFT calculations, the design and optimization of NRR diatomic catalysts can be effectively explored, particularly in the rapid screening of candidates, showcasing broad prospects.

In this study, we selected 18 different combinations of transition metal atoms to serve as active sites in the DAC model. By cross-combining these atoms, we designed a total of 171 catalysts with the objective of thoroughly exploring their catalytic potential. We then chose 6-nitrogen-doped graphene (N_6_G) as the substrate and abbreviated these diatomic catalysts as TM_1_-TM_2_@N_6_G. The structural and performance parameters of the catalysts were derived from a chemical property database and simple DFT calculations, which were used to assess the performance of 25 groups of TM_1_-TM_2_@N_6_G catalysts. For machine learning screening, we employed four indicators: catalyst binding energy, adsorption energy of N_2_ on the catalyst, the energy of the first hydrogenation step, and ammonia dissociation energy. We utilized three machine learning algorithms for comparative screening: the random forest algorithm, k-nearest neighbor regression, and a decision tree. Ultimately, we successfully screened four TM_1_-TM_2_@N_6_G catalysts that demonstrated high activity and selectivity during N_2_ activation. The stability of these catalysts, as well as the extent of N_2_ activation and the reaction energy barrier, were evaluated using ab initio molecular dynamics (AIMD), Bader charge analysis, projected density of states (PDOS), and energy band analysis. The results indicate that the double-transition metal atoms anchored to the N_6_G monolayer can effectively reduce N_2_ to NH_3_, showcasing their broad application potential in nitrogen reduction reactions.

## 2. Structure and Discussion

### 2.1. TM_1_-TM_2_@N_6_G Catalyst Structure and Stability

Previous studies have extensively explored the characteristics and performance of nitrogen-doped graphene (NG) as a catalyst substrate [34]. NG is renowned for its excellent stability, maintaining superior catalytic performance under harsh conditions [35]. Furthermore, NG features tunable active site coordination structures [36,37]. According to prior research, hexa-nitrogen coordination structures have been found to be beneficial for dual-Atom catalysts (DACs) by facilitating direct charge transfer between two metal atoms connected via a metal–metal bond. Additionally, tri-nitrogen coordination structures have demonstrated exceptional electron transfer properties and catalytic activity in DACs [35,38]. Therefore, this study focuses on investigating the micro-coordination structure (N_6_G), wherein a single metal atom is coordinated by three nitrogen atoms.

**Figure 1:**
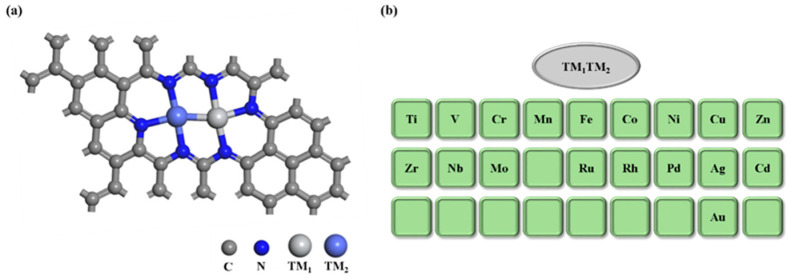
(**a**) Structure of TM_1_-TM_2_@N_6_G. (**b**) The transition metal elements selected for this study.

In the selection of metal atoms, those from the 3d, 4d, and 5d transition series, as well as main group metals, exhibit promising potential as active sites in catalysts. Thus, we chose 18 transition metal atoms for the TM_1_-TM_2_@N_6_G model, as illustrated in Figure 1a. To succinctly describe the catalysts’ structure and type, a series of dual-atom catalysts (DACs) is denoted as TM_1_-TM_2_@N_6_G, as shown in Figure 1b. Here, TM1 and TM2 represent transition metal atoms anchored on the N_6_G surface, including Ti, V, Cr, Mn, Fe, Co, Ni, Cu, Zn, Zr, Nb, Mo, Ru, Rh, Pd, Ag, Cd, and Au. Considering the structural diversity and complexity of TM_1_-TM_2_@N_6_G, these 18 metal atom combinations yield a total of 171 possible TM_1_-TM_2_@N_6_G catalysts. However, conducting DFT calculations for all 171 catalysts would be computationally intensive and time-consuming, resulting in inefficient use of resources. Therefore, we randomly selected 25 catalysts for performance exploration, serving as the dataset for subsequent machine learning (ML) screening.

**Figure 2:**
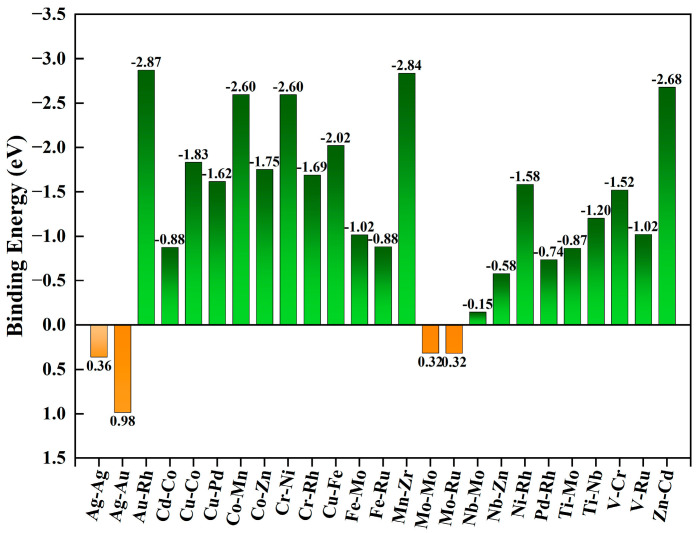
Binding energies of 25 TM_1_-TM_2_@N_6_G.

The achievement of catalytic performance hinges on the structural stability of the catalyst. Only when the catalyst structure is stable can it effectively promote chemical reactions, thereby attaining the desired catalytic activity and selectivity. For the selected 25 TM_1_-TM_2_@N_6_G structures, after structural optimization calculations, their formation energies ($E_{G}$) were computed using the formation energy formula, as depicted in Figure 2. A negative formation energy indicates that these structures are thermodynamically stable, as they can form spontaneously without requiring external energy. Conversely, a positive formation energy value indicates that these structures require additional external energy to form, rendering them thermodynamically unstable. Through an in-depth exploration of the structural properties of TM_1_-TM_2_@N_6_G catalysts, significant differences have been identified. As illustrated in Figure 2, the 21 groups of catalysts exhibit robust stability, primarily attributed to their negative formation energies. Notably, the Au-Rh@N_6_G and Mn-Zr@N_6_G catalysts demonstrate exceptionally high binding energies of −2.87 eV and −2.84 eV, respectively, further highlighting their stability advantages. Four groups of catalysts display weaker stability, with formation energies approaching or even reaching positive values. Among these, the Mo-Mo@N_6_G and Mo-Ru@N_6_G catalysts, although their metal atoms remain bonded to the N_6_G substrate without complete detachment, possess certain research potential. However, their positive binding energies indicate a relatively weaker stability. In contrast, the Ag-Ag@N_6_G and Ag-Au@N_6_G catalysts not only exhibit positive formation energies but also show evident structural instability, as their metal atoms have detached from the N_6_G substrate. Consequently, they are unsuitable for subsequent research as stable catalysts. In summary, it can be concluded that the synthesis of stable TM_1_-TM_2_@N_6_G catalysts is entirely feasible.

### 2.2. Computational Screening of NRR Catalytic Candidates

**Figure 3:**
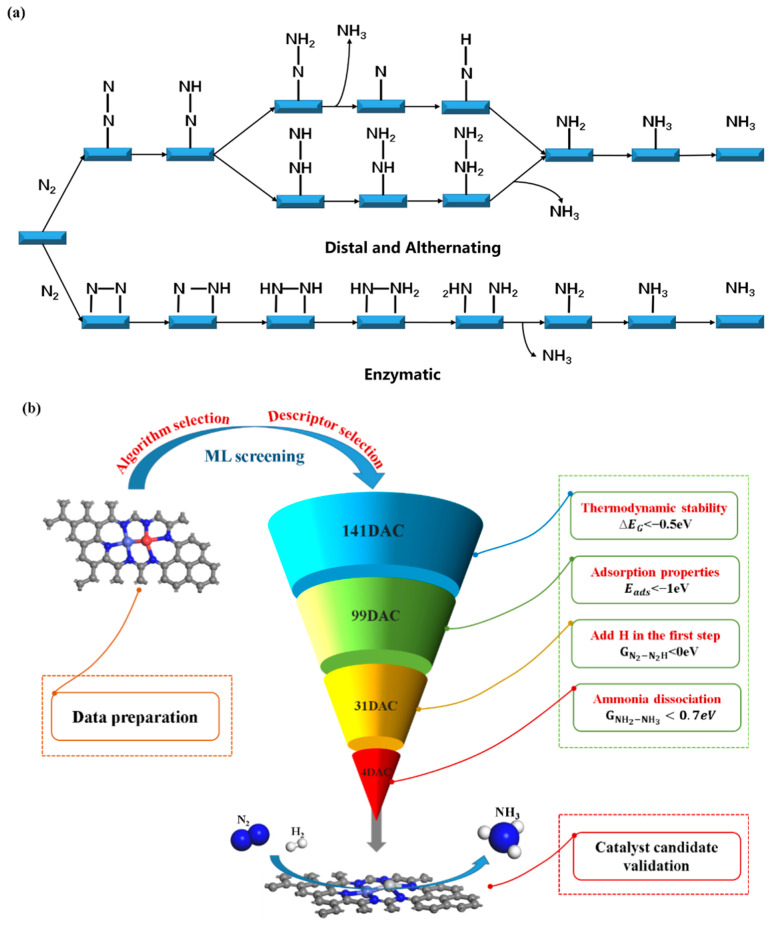
(**a**) The reaction mechanism of NRR and (**b**) the screening criteria of ∆$E_{G}$, $E_{ads}$, $∆G_{N_{2}-N_{2}H}$, $∆G_{{NH}_{2}-{NH}_{3}}$ and the ML screening process.

The chemical reaction process of NRR is highly intricate, encompassing various potential reaction mechanisms such as distal, alternating, and enzymatic reactions (Figure 3a). Traditional research methodologies often involve utilizing density functional theory to comprehensively investigate all feasible reaction intermediates, aiming to pinpoint potential reaction pathways and theoretical rate-determining steps. However, this approach entails substantial computational expenses, rendering it particularly inefficient for large-scale catalyst screening endeavors. To strike a balance between computational cost and efficiency in catalyst screening, we have adopted a simplified approach: selecting specific descriptors to offer a brief description of the NRR reaction. These descriptors may encompass pivotal physical or chemical attributes, including electron affinity, atomic charge distribution, and the stability of reaction intermediates, among others. By leveraging these descriptors, we can promptly assess the potential activity and selectivity of diverse catalysts in the NRR process, thereby strategically diminishing the pool of candidate catalysts necessitating detailed computations. This refined approach facilitates a more efficient evaluation of catalyst performance, optimizing the selection procedure for efficacious NRR catalysts.

The structural stability of a catalyst is a fundamental prerequisite for its functionality. In the NRR process, if the TM_1_-TM_2_@N_6_G catalyst lacks stability, it becomes irrelevant to the reaction. Therefore, we consider the catalyst binding energy (∆$E_{G}$) as a crucial descriptor. Furthermore, when the TM_1_-TM_2_@N_6_G catalyst participates in the NRR reaction, its chemisorption of N_2_ is of utmost importance. Effective chemisorption can fully activate the inert N≡N triple bond, reducing the required reaction energy between the N≡N bonds, lowering the reaction barrier, and increasing the frequency of effective collisions between the reactant and the catalyst. Consequently, we consider the adsorption energy ($E_{ads}$) of N_2_ as the second descriptor. Additionally, an examination of all reaction intermediates in the NRR process reveals that the initial step of adding H to form N_2_H (N_2_ + H = N_2_H) and the final step of H desorption to form NH_3_ (*NH_2_ + H = *NH_3_) are consistently present, regardless of the NRR reaction mechanism. In NRR, due to the inertia of the N≡N triple bond, the first H addition step requires a significant reaction energy ($∆G_{N_{2}-N_{2}H}$) to break this bond, which is theoretically likely to be the rate-determining step in NRR. Therefore, we regard it as the third descriptor, reflecting the reaction’s difficulty. Ammonia dissociation is also critical in the NRR reaction. The nitrogen atom forms four orbitals through sp^3^ hybridization, three of which are half-filled; thus, its hybridization method is sp^3^. When N atoms adsorb H atoms to form NH_2_, only one sp^3^ hybrid orbital remains, making it prone to adsorption on the catalyst surface to form a stable structure. As a result, the formation and desorption of *NH_2_ to *NH_3_ on the catalyst require substantial free energy [39]. However, DFT calculations of the selected 25 catalysts revealed that the direct transition from *N_2_ to *N_2_H is rare; instead, it often occurs through a side path from *NH_2_ to NH_3_. Many catalysts exhibit better activity via this lateral pathway without undergoing the *NH_2_ to *NH_3_ process. Therefore, we choose the energy change of *NH_2_-NH_3_ ( $∆G_{{NH}_{2}-{NH}_{3}}$) as the fourth descriptor. Numerous studies have confirmed that $∆G_{{NH}_{2}-{NH}_{3}}$ and $∆G_{{NH}_{2}-{NH}_{3}}$ generally have large positive values throughout the reduction process for most catalysts, regardless of the reaction mechanism [40,41]. Thus, using $∆G_{N_{2}-N_{2}H}$ and $∆G_{{NH}_{2}-{NH}_{3}}$ as activity descriptors can effectively reflect the catalyst’s performance. By employing these four descriptors, the screening efficiency can be significantly enhanced without compromising screening accuracy.

Based on the aforementioned analysis, we devised a two-step screening process: in the initial phase, ∆$E_{G}$ and $E_{ads}$ served as the primary screening criteria to eliminate catalysts with insufficient stability and poor N_2_ activation. In the subsequent phase, highly active catalysts were screened using $∆G_{N_{2}-N_{2}H}$ and $∆G_{{NH}_{2}-{NH}_{3}}$. To identify the optimal DACs, we established the following criteria for each descriptor (Figure 3b): (i) ∆$E_{G}$ should be less than −0.5 eV, ensuring robust stability; (ii) $E_{ads}$ should be less than −1 eV, corresponding to the degree of chemisorption and activation of N_2_; (iii) $∆G_{N_{2}-N_{2}H}$ should not exceed 0 eV, ensuring that the cleavage of the N≡N triple bond during NRR is a spontaneous process, thus significantly reducing the reaction’s complexity; (iv) $∆G_{{NH}_{2}-{NH}_{3}}$ should not surpass 0.70 eV, which, according to the Arrhenius equation, suggests that the produced NH_3_ can be swiftly removed. Furthermore, these four descriptors can be tailored to suit the specific requirements of individual research endeavors.

Firstly, the structures and reaction intermediates of 25 TM_1_-TM_2_@N_6_G were investigated using the DFT calculation method, from which the four descriptor parameters—∆$E_{G}$, $E_{ads}$, $∆G_{N_{2}-N_{2}H}$, and $∆G_{{NH}_{2}-{NH}_{3}}$—for each of the 25 TM_1_-TM_2_@N_6_G systems were obtained.

**Figure 4:**
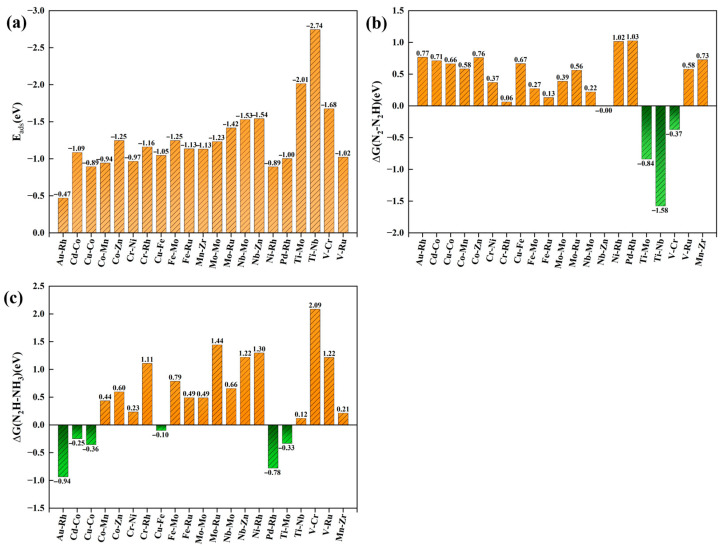
(**a**) $E_{ads}$, (**b**) $∆G_{N_{2}-N_{2}H}$, and (**c**) $∆G_{{NH}_{2}-{NH}_{3}}$ energy of 21 groups of DACs.

The calculated four descriptor parameters are presented in Table S1, where the adsorption energy is denoted by the maximum adsorption energy observed among the various adsorption structures. As illustrated in Figure 4, the graphene-based double-transition metal atom DACs exhibit outstanding performance in terms of $E_{ads}$, $∆G_{N_{2}-N_{2}H}$, and $∆G_{{NH}_{2}-{NH}_{3}}$. From Figure 4a, it is evident that the adsorption energy of N_2_ on the catalyst is less than 0, with a maximum adsorption energy of −2.01 eV. This indicates that the graphene-based DACs possess strong N_2_ adsorption capabilities, thereby establishing a foundation for subsequent N_2_ activation. Figure 4b shows that in the first hydrogenation step, three catalyst groups, namely Ti-Mo, Ti-Nb, and V-Cr, exhibit negative energy changes, while 16 groups have energy changes of less than 0.8 eV. This further indicates that DACs effectively activate the N≡N bond, reducing its bond energy and resulting in a low energy barrier for the initial hydrogenation activation reaction, which facilitates the reaction under milder conditions. As illustrated in Figure 4c, during the NH_3_ dissociation process, six out of the 21 catalyst groups exhibit spontaneous NH_3_ desorption, suggesting that these DACs possess good dissociation ability.

These four descriptor parameters serve as the target values for training and testing the ML model. Subsequently, input features are selected, which are crucial for constructing the ML model and identifying the intrinsic correlations within the input data. The chosen model features should reflect the atomic and electronic properties of various TM atoms, considering their interaction with N_6_G. To ensure the efficiency of the ML method, these features should be readily obtainable through simple DFT calculations or direct database queries. Based on the aforementioned principles, eight distinct physicochemical properties were selected to characterize the key intermediate reactions in the TM_1_-TM_2_@N_6_G catalytic NRR process for the ML model. The electronic structure of TM metal atoms is described by metal negativity (p), electron affinity (EA), first ionization energy (IE), and d electron number (N_d_). Additionally, considering the unique structure of TM_1_-TM_2_@N_6_G and the interaction between TM atoms and N_6_G, we included the sum of the van der Waals radii (R) of TM atoms, the atomic mass (M) of TM atoms, and the atomic radii of the two TM atoms. Ultimately, we compiled 23 input features, as presented in Table 1. It is important to note that high correlation between features can adversely affect the performance of the ML model. Therefore, we utilized the Pearson correlation coefficient matrix to assess the correlation between feature pairs, eliminating features with high correlation among the input features and retaining those that are highly correlated with the output descriptor. With the assistance of ML methods, the catalytic performance of all TM combinations can be rapidly determined, enabling the exploration of interaction modes with key intermediates and the screening of ideal catalysts.

Based on the aforementioned conditions, we utilized three ML regression algorithms: RFR, KNR, and DT for ∆$E_{G}$, $E_{ads}$, $∆G_{N_{2}-N_{2}H}$, and $∆G_{{NH}_{2}-{NH}_{3}}$, respectively, to establish ML models for analysis. Each model was optimally tuned using a large number of parameters. We evaluated the models using R^2^ and RMSE. To prevent overfitting during the tuning process, we included performance evaluations for both the training set and the test set. As shown in Table 2, the performance of the models based on the KNR and DT algorithms is significantly inferior to that of the RFR algorithm model. Therefore, we selected the RFR algorithm as the model algorithm for subsequent ML tasks.

The trained RFR model was used to predict the ∆$E_{G}$, $E_{ads}$, $∆G_{N_{2}-N_{2}H}$, and $∆G_{{NH}_{2}-{NH}_{3}}$ of the remaining 146 TM_1_-TM_2_@N_6_G structures. The input dataset consisted of the atomic and electronic properties of 18 TM atoms, enabling us to efficiently screen highly active catalysts from 171 TM_1_-TM_2_@N_6_G structures, thereby reducing computational costs. In this study, DFT calculations for ∆$E_{G}$, $E_{ads}$, $∆G_{N_{2}-N_{2}H}$, and $∆G_{{NH}_{2}-{NH}_{3}}$ for 25 TM_1_-TM_2_@N_6_G structures alone took approximately 1200 h. In contrast, the training and prediction of the ML model, along with hundreds of tests of the three algorithms against four descriptors, were completed in less than 20 h. This demonstrates that the DFT-ML coupling approach used significantly reduces the computational cost. As a result, the research and screening efficiency for numerous catalysts has been improved.

**Figure 5:**
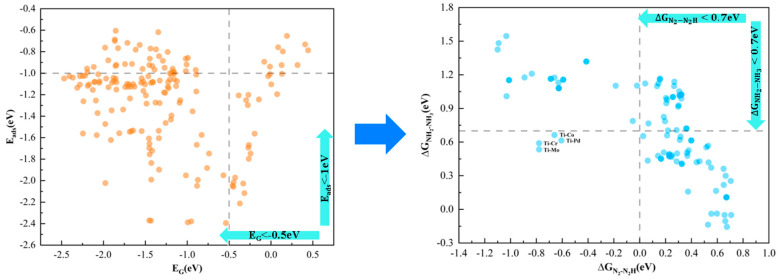
ML screened 171 TM_1_-TM_2_@N_6_G catalysts.

Catalyst screening was conducted based on the descriptor parameters ∆$E_{G}$, $E_{ads}$, $∆G_{N_{2}-N_{2}H}$, and $∆G_{{NH}_{2}-{NH}_{3}}$ obtained from the ML model. As shown in Figure 5, the first step involved excluding catalysts with poor stability and weak N_2_ adsorption capacity. As a result, 99 candidate catalysts were screened from 171 species, all of which exhibited good stability and high N_2_ adsorption. In the second step, the 99 catalysts were further screened for activity, and it was found that only 4 out of the 99 catalysts met the set conditions and could achieve a low reaction energy pathway. Ultimately, we identified four highly active candidate catalysts: Ti-Mo@N_6_G, Ti-Cr@N6G, Ti-Pd@N_6_G, and Ti-Co@N_6_G.

### 2.3. Performance Exploration of TM_1_-TM_2_@N6G Candidates

**Figure 6:**
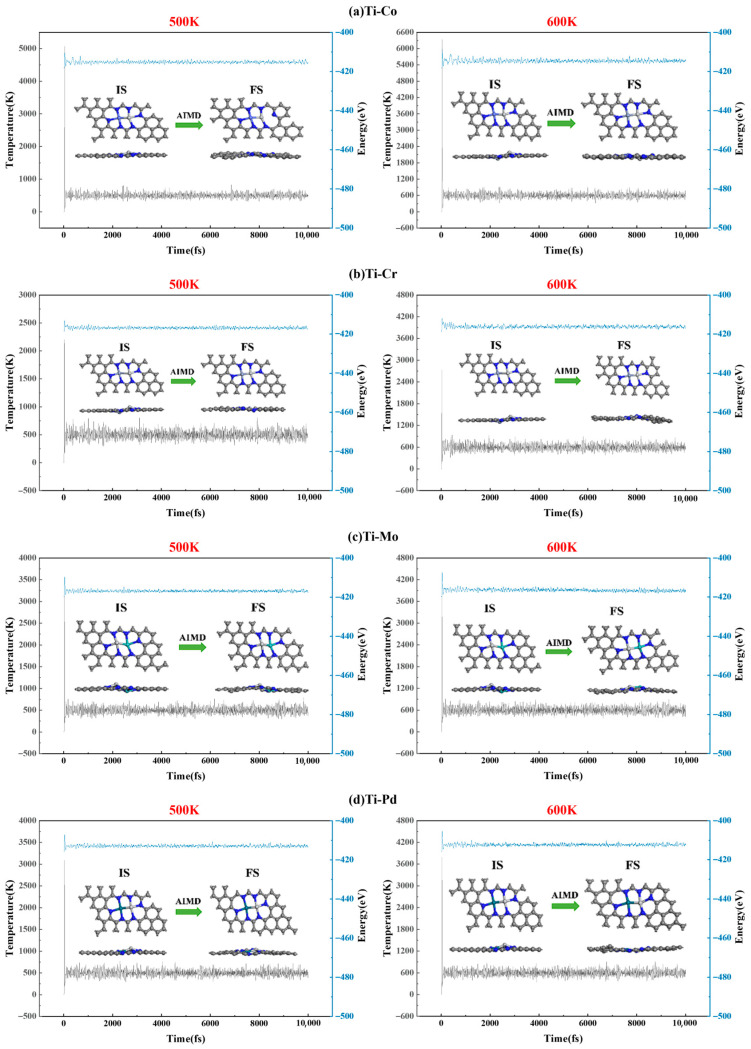
Changes in energy and temperature during AIMD simulation of (**a**) Ti-Co, (**b**) Ti-Cr, (**c**) Ti-Mo, and (**d**) Ti-Pd.

To further investigate the catalytic performance of the four screened TM_1_-TM_2_@N_6_G catalysts, AIMD simulations were conducted at 500 K and 600 K, respectively, to evaluate their stability using 1 fs time steps over a total simulation time of 10 ps. As shown in Figure 6a–d, the energy and structure of the four TM_1_-TM_2_@N_6_G catalysts remained stable at high temperatures of 500 K and 600 K, with the two TM atoms firmly bound to the six N atoms on the catalyst without any significant structural distortion. This demonstrates that the four TM_1_-TM_2_@N_6_G catalysts exhibit thermal stability and are capable of withstanding high temperatures.

To assess the activation mechanism and explore N_2_ adsorption on the four catalysts, the presence of two active sites in the bimetallic atom catalyst introduced diversity in N_2_ adsorption, revealing new activation possibilities and reaction pathways. As evident from Figure S1, there are three forms of N_2_ adsorption on the catalyst surface: single adsorption structure, bridge adsorption structure, and parallel adsorption structure. Detailed information, including the calculated adsorption energy ($E_{ads}$), the length of the N≡N bond under different adsorption structures, and the Bader charge, is presented in Table 3. The adsorption energy is linearly related to the adsorption strength, with a more negative adsorption energy indicating a stronger adsorption intensity of the corresponding adsorbate. According to Table 3, the N_2_ adsorption energies for Ti-Co-Parallel, Ti-Co-Single, Ti-Mo-Parallel, Ti-Cr-Parallel, and Ti-Pd-Single were −0.733, −0.748, −2.014, −2.020, and −1.134 eV, respectively. It was observed that the four candidate catalysts did not exhibit a bridge adsorption structure, and the single adsorption structure and parallel adsorption structure demonstrated a stronger preference for N_2_ adsorption. Among these, Ti-Mo-Parallel and Ti-Cr-Parallel showed a particularly strong adsorption trend.

**Figure 7:**
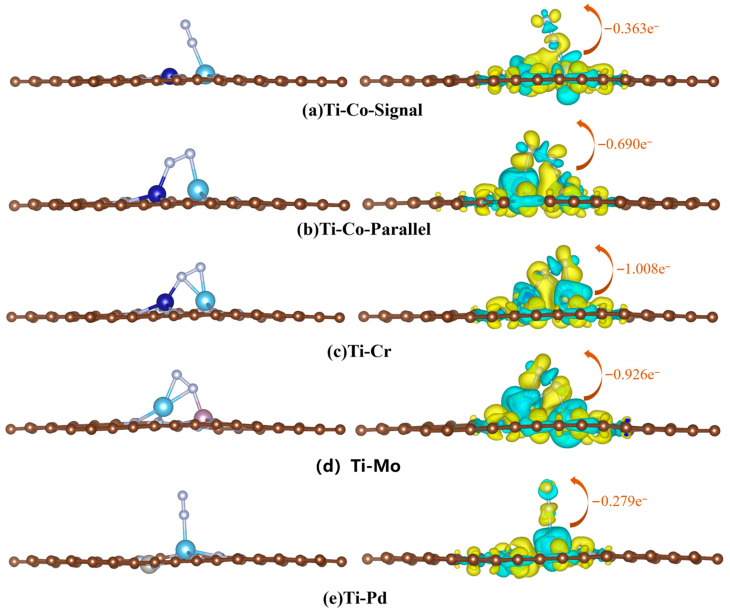
Diagram of the adsorption structure and the corresponding differential charge density of the candidate DACs.

The activation mechanism of N_2_ by the four candidate catalysts was further revealed, and the differential charge density map of the adsorption structure was obtained. As shown in Figure 7, the cyan area represents charge loss, and the yellow area represents charge accumulation. According to the differential charge density diagram, there is a large amount of electron transfer between the active site and N_2_ when N_2_ is adsorbed on the surface. Significant charge loss occurs at the N≡N bond, which weakens the N≡N interaction and realizes the activation of N_2_. It can be seen that the parallel adsorption structure has more advantages in activating N_2_ molecules, and the Ti-Mo-Parallel and Ti-Cr-Parallel surfaces obtain charges of 1.008 and 0.926e^−^, which are more dense than those of the single adsorption structure, indicating that the parallel adsorption structure is more conducive to the transfer of charge from the catalyst to the adsorbate. The corresponding N≡N bond length (between 1.145 Å and 1.249 Å) varies with the number of transferred electrons. The increase in N≡N bond length after adsorption further confirmed the effective activation of N_2_.

**Figure 8:**
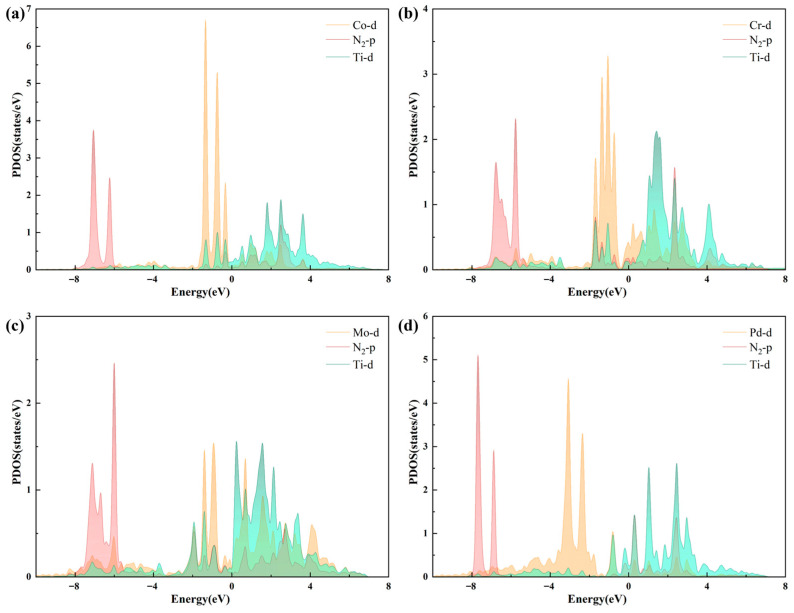
The projected density of states (PDOS) of (**a**) Ti-Co, (**b**) Ti-Cr, (**c**) Ti-Mo, and (**d**) Ti-Pd.

To reveal the microscopic electron distribution and density of states of the catalyst, projected density of states (PDOS) calculations and energy band diagrams of the catalyst were performed, with the Fermi level set to 0. As shown in Figure 8, there is evident hybridization and coupling between the p orbital of the N_2_ molecule and the d orbital of the active site in the catalyst. The significant overlap of the d and p orbitals indicates a strong bond between the N_2_ molecules and the substrate, which is advantageous for the capture of N_2_. The interaction between N_2_ and TM_1_-TM_2_@N_6_G follows the well-established donation–back-donation mechanism. Specifically, electrons from the filled π orbitals of N_2_ are donated to the unoccupied d orbitals of the TM metal atom, while the occupied d orbitals of the TM atom back-donate electrons into the antibonding π* orbitals of N_2_. This synergistic donation and back-donation interaction facilitates the activation of the N≡N bond, confirming that the four catalysts exhibit strong adsorption and high activity towards N_2_.

**Figure 9:**
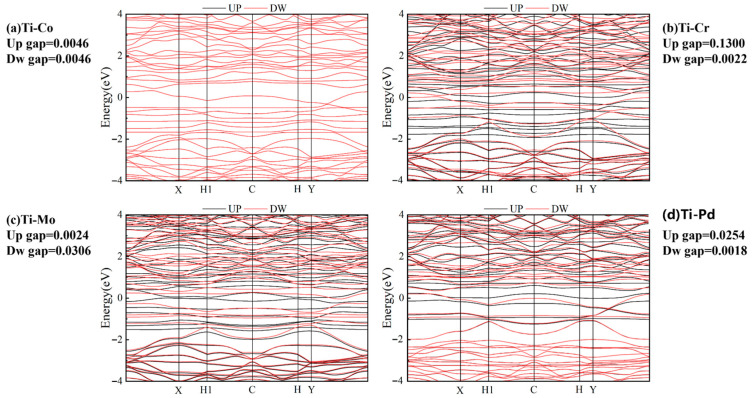
Band structure of (**a**) Ti-Co, (**b**) Ti-Cr, (**c**) Ti-Mo, and (**d**) Ti-Pd. Spin-up band gap and spin-down band gap are labeled separately.

As illustrated in Figure 9a–d, the four catalysts, namely Ti-Co@N_6_G, Ti-Cr@N_6_G, Ti-Mo@N_6_G, and Ti-Pd@N_6_G, all exhibit narrow band gaps. Among them, Ti-Pd@N_6_G has the narrowest spin-down band gap of 0.0018 eV, while Ti-Mo@N_6_G displays the narrowest spin-up band gap of 0.0024 eV. A narrower band gap signifies a higher electron transfer and transmission rate on the catalyst surface, resulting in faster internal electron transitions and, consequently, enhanced catalyst activity.

**Figure 10:**
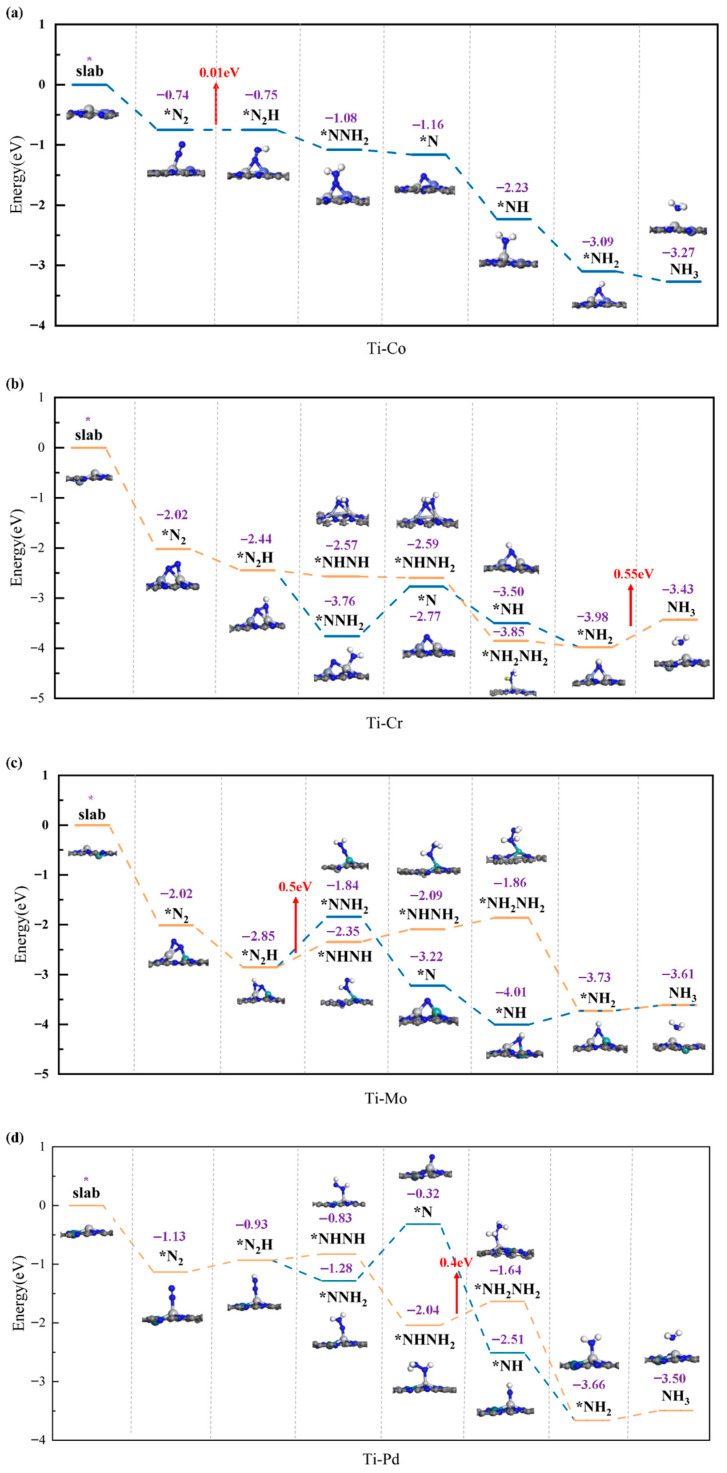
(**a**) Ti-Co, (**b**) Ti-Cr, (**c**) Ti-Mo, and (**d**) Ti-Pd free energy distribution and reaction energy (* The symbol is used to indicate the intermediates adsorbed on the surface of the catalyst).

To comprehensively assess the activity of the catalyst and verify its validity and accuracy, a detailed exploration of the NRR mechanism was conducted for the four catalyst structures. During the screening process, only intermediates with stable structures were retained. The NRR mechanism diagram and the corresponding intermediate structure diagrams are presented in Figure 10a–d. All four catalysts demonstrate excellent catalytic performance. Specifically, Ti-Co@N_6_G exhibits only one favorable NRR pathway. In contrast, Ti-Cr@N_6_G, Ti-Mo@N_6_G, and Ti-Pd@N_6_G possess two favorable pathways, which can be categorized as the far-end and alternating paths:

*N_2_ → *NNH → *NNH_2_ → *N → *NH → *NH_2_ → *NH_2_+1/2H_2_ → NH_3_(g)

*N_2_ → *NNH → *NHNH → *NHNH_2_ → *NH_2_ → *NH → *NH_2_ → *NH_2_+1/2H_2_ → NH_3_(g).

From Figure 10b–d, it can be seen that the reaction energies of the alternating paths in Ti-Cr@N_6_G, Ti-Mo@N_6_G, and Ti-Pd@N_6_G are much smaller than those of the distal paths, and the reaction energies are 0.55 eV, 0.5 eV, and 0.4 eV, respectively. There is only a distal path in the Ti-Co@N_6_G catalyst, and the best NRR path reaction energy is 0.01 eV, which can realize the spontaneous catalysis of the NRR process. In addition, it is worth noting that it can be observed from Figure 10a–d that the first hydrogenation reaction of nitrogen can occur without or with very little external energy input, which is not common in previous related studies. This phenomenon further reveals that the adsorbed N_2_ molecules are highly activated by the catalyst, and the interaction between N≡N bonds is greatly reduced, which further improves the catalytic efficiency.

## 3. Computational Details

The density functional theory (DFT) calculations in this study were performed using the Vienna Ab initio Simulation Package (VASP) [42]. The interaction between electrons and ions was described using the Projector Augmented Wave (PAW) method. The exchange correlation was described by the Generalized Gradient Approximation (GGA) in the Perdew–Burke–Ernzerhof (PBE) parameterization [43]. The weak van der Waals interaction between the substrate and the adsorbate was corrected using the DFT-D_3_ method [44]. The cut-off energy was set to 400 eV. A vacuum layer of 15 Å was established to prevent periodic repetitive effects during structural relaxation. The transition metal diatomic catalyst model was created by doping six nitrogen atoms onto a 4 × 4 × 1 graphene supercell and anchoring the transition metal atom pair. The first Brillouin zone was sampled using the Monkhorst–Pack method, with the Kpoints file set to 3 × 4 × 1. The energy convergence criterion was set to 1 × 10^−6^ eV. The charge transfer and distribution between the intermediate and the substrate were quantified using Bader charge analysis [45].

The thermodynamic stability of TM_1_-TM_2_@N_6_G was investigated through AIMD calculations at 600 K, with a 10 ps simulation duration and 1 fs time steps. The data generated by VASP were analyzed using VASPKIT [46]. The s, d orbital projected density of states (PDOS) of the active center metal atoms and the p orbital PDOS of the N_2_ small molecule were calculated and plotted to describe the electron distribution within each orbital. These charts were used to analyze atom-to-atom interactions and provide insights into the details of chemical bonds. To ensure the accuracy of the results, a NEDOS value of 4000 was employed in this study.

To demonstrate the feasibility and stability of the diatomic catalyst, the binding energies ($E_{G}$) of TM_1_-TM_2_@N_6_G were calculated:

(1) $$E_{G}=E_{slab}-E_{N-graphite}-\mu_{M}-\mu_{X},$$

$E_{slab}$ and $E_{N-graphite}$ are the total energies of TM_1_-TM_2_@N_6_G and N-doped graphite, respectively. $\mu_{M}$ and $\mu_{X}$ represent the chemical potential of each metal element, respectively.

To evaluate the stability of TM_1_-TM_2_@N_6_G adsorption to N_2_, the adsorption energies ($E_{ads}$) of NRR intermediates were calculated.

(2) $$E_{ads}=E_{total}-E_{Rel}-E_{sub}$$

$E_{sub}$ and $E_{Rel}$ represent the energy of the substrate and the relaxed adsorbate, respectively. The energy of the adsorption system is denoted by $E_{total}$. The larger the absolute value of the adsorption energy ($E_{ads}$), the more stable the adsorption structure is.

To study the reaction mechanism of N_2_ and H_2_ converting to NH_3_ in TM_1_-TM_2_@N_6_G diatomic catalysts, the change in the intermediate reaction energy at each step, denoted as ∆G, was calculated.

(3) $$\;∆G=E_{aft}-E_{bef}-E_{ecp},$$

∆G denotes the energy difference of the intermediate before and after the reaction. $E_{bef}$ and $E_{aft}$ represent the energy of the intermediate before and after the reaction, and $E_{ecp}$ represents the energy change in the elements involved in the reaction. This value does not represent a true activation energy derived from a transition state calculation but rather serves as an effective descriptor to assess catalytic trends.

Three different supervised machine learning algorithms were utilized in the machine learning process: the random forest algorithm (RFR) [47], k-nearest neighbor regression (KNR) [48], and decision tree (DT) [49]. These algorithms were implemented in the Python3 environment using the open-source Scikit-learn and PyTorch packages [50]. The input data, generated by DFT computations and the database, was trained and divided into a training set and a test set at a ratio of 8:2. All data was normalized during both training and prediction. The performance of each ML model was evaluated using two indicators: root mean square error (RMSE) and the coefficient of determination (R^2^), which are expressed as follows:

(4) $$RMSE=\sqrt{\frac{1}{n}\sum_{i=1}^{n}{\left(p_{i}-y_{i}\right)}^{2}}$$

(5) $$R^{2}=1-\frac{\sum_{i=1}^{n}{\left(p_{i}-y_{i}\right)}^{2}}{\sum_{i=1}^{n}{\left(p_{i}-\bar{y_{i}}\right)}^{2}}$$

where $y_{i}$ represents the true value and $p_{i}$ represents the model’s predicted value. For an accurate model, the RMSE should ideally be as close to 0 as possible, and the R^2^ score should be as close to 1 as possible.

## 4. Summary and Conclusions

In summary, in this study, the performance of 171 different DACs catalyzed by 17 transition metal elements in pairs was systematically investigated by combining DFT computation and machine learning methods. In this study, 25 randomly selected DACs were calculated by DFT to obtain key intermediates and their free energy changes, and combined with the physical parameters of the corresponding metal atoms constituting DACs, they were used as the training set for machine learning research for effective model training. It was found that the model based on the RFR algorithm had the highest prediction accuracy for the adsorption energy of reaction intermediates (R^2^ > 0.9, RMSE < 0.18). Based on the constraints of catalyst binding energy, nitrogen adsorption energy, the first hydrogenation step, and ammonia dissociation, four candidate high-activity catalysts were screened out. Through performance exploration, it is found that the synergistic effect of the TM_1_-TM_2_@N_6_G catalyst and two TM atoms breaks the inherent limitation of the linear relationship between intermediates, promotes the activation and adsorption of N_2_, and has great potential for the deep reduction of N_2_. The four candidate catalysts screened showed excellent stability, significant affinity, and high selectivity for N_2_. Among them, Ti-Cr@N_6_G, Ti-Mo@N_6_G, and Ti-Pd@N_6_G catalysts have two different reaction paths, and the lowest reaction energies appear on the alternating path, which are 0.55 eV, 0.5 eV, and 0.4 eV, respectively. The reaction of Ti-Co@N_6_G only follows the distal path, and its reaction energy is extremely low, only 0.01 eV, which means that Ti-Co@N_6_G can spontaneously catalyze during the NRR process. The method proposed in this paper to determine the performance of catalysts by descriptors by combining ML and DFT greatly reduces the cost of calculation and can quickly and accurately determine the highly active candidate catalysts in a class of catalysts, thus providing a new perspective for the design of NRR catalysts with excellent catalytic performance.

## Figures and Tables

**Table 1:** Machine learning initial input features.

Input Feature Value	Symbol	Input Feature Value	Symbol
The number of d orbital electrons of metal A	N_dA_	The first ionization energy of metal B	IE_B_
The number of d orbital electrons of metal B	N_dB_	The absolute value of the first ionization energy between metals	IE
The absolute value of the number of d orbital electrons between metals	N_d_	The electron affinity energy of metal A	EA_A_
The sum of the number of d orbital electrons between metals	N_dAB_	The electron affinity energy of metal B	EA_B_
Electronegativity of metal A	p_A_	The absolute value of the electron affinity between metals	E_A_
Electronegativity of metal B	p_B_	The atomic radius of metal A	R_A_
The absolute value of the difference in electronegativity between metals	p_A−B_	The atomic radius of metal B	R_B_
The sum of electronegativity between metals	p_A+B_	The sum of the atomic radii of the center of the metal	R_A+B_
The number of s orbital electrons of metal A	N_sA_	The atomic mass of metal A	M_A_
The number of s orbital electrons of metal B	N_sB_	The atomic mass of metal B	M_B_
The absolute value of the number of s orbital electrons between metals	N_s_	The atomic mass of metals A, B	M_A+B_
The first ionization energy of metal A	IE_A_		

**Table 2:** Comparison of three ML regression algorithms: RFR, KNR, and DT.

ML Algorithm	Evaluation Criterion	$E_{G}$/eV	$E_{ads}$/eV	$∆G_{N_{2}-N_{2}H}$/eV	$∆G_{{NH}_{2}-{NH}_{3}}$/eV
RFR	R^2^	0.9054	0.9129	0.9233	0.9095
	RMSE	0.1698	0.1219	0.1664	0.1644
DT	R^2^	0.8171	0.7071	0.6310	0.8417
	RMSE	0.4475	0.4613	0.5314	0.3739
KNR	R^2^	0.7777	0.7655	0.6920	0.8164
	RMSE	0.3504	0.2134	0.3033	0.2752

**Table 3:** Stable adsorption structure details of candidate catalysts.

	N≡N/Å			Bader/e^−^			Eads/eV		
	Bridge	Parallel	Single	Bridge	Parallel	Single	Bridge	Parallel	Single
Ti-Co	—	1.214	1.145	—	−0.690	−0.363	—	−0.733	−0.748
Ti-Mo	—	1.245	—	—	−1.008	—	—	−2.014	—
Ti-Cr	—	1.249	—	—	−0.926	—	—	−2.020	—
Ti-Pd	—	—	−1.138	—	—	−0.279	—	—	−1.134

## Data Availability

Data will be made available on request.

## Supplementary Materials

The following supporting information can be downloaded at https://www.mdpi.com/article/10.3390/molecules30204131/s1, Table S1: Contains four descriptor parameters. Figure S1: Schematic diagram of common adsorption structures of NRR. Figure S2: Comparision between DFT calculation results and RFR predictions for (a) ∆$E_{G}$, (b) $E_{ads}$, (c) $∆G_{N_{2}-N_{2}H}$, (d) $∆G_{{NH}_{2}-{NH}_{3}}$.

## Abbreviations

The following abbreviations are used in this manuscript:

NRR	Nitrogen reduction reaction
ML	Machine learning
DFT	Density functional theory
SACs	Single-atom catalysts
DACs	Dual-atom catalysts
N_6_G	6-nitrogen-doped graphene
AIMD	Ab initio molecular dynamics
PDOS	Projected density of states
VASP	Vienna Ab initio Simulation Package
PAW	Projector Augmented Wave
GGA	Generalized Gradient Approximation
PBE	Perdew–Burke–Ernzerhof
RFR	Random Forest Algorithm
KNN	K-nearest Neighbor Regression
DT	Decision Tree
RMSE	Root mean square error
R^2^	Coefficient of determination
