# Supplementary material for: Machine Learning-Assisted DFT Screening of Nitrogen-Doped Graphene Diatomic Catalysts for Nitrogen Reduction Reaction
Source: Molecules. 2025 Oct 20;30(20):4131. doi: 10.3390/molecules30204131 (PMC12565872; doi:10.3390/molecules30204131)
Supplement: Supplementary file 1 [file molecules-30-04131-s001.zip › molecules-3840497-supplementary.pdf]

molecules

MDPI

Supporting Information

Table S1. Contains four descriptor parameters

The type of active site	E_G/eV	E_{ads}/eV	$\Delta G_{N_2-N_2H}$/eV	$\Delta G_{NH_2-NH_3}$/eV
Ag-Ag	0.3621	-	-	-
Ag-Au	0.9841	-	-	-
Au-Rh	-2.8716	-0.470	0.767	-0.935
Cd-Co	-0.8758	-0.928	0.711	-0.248
Cu-Co	-1.8309	-0.878	0.664	-0.355
Cu-Pd	-1.6173	-	-	-
Co-Mn	-2.5966	-1.097	0.582	0.435
Co-Zn	-1.7514	-1.247	0.760	0.596
Cr-Ni	-2.5963	-0.967	0.369	0.234
Cr-Rh	-1.6885	-1.165	0.062	1.109
Cu-Fe	-2.0221	-1.046	0.666	-0.100
Fe-Mo	-1.016	-1.247	0.272	0.787
Fe-Ru	-0.8827	-1.014	0.131	0.491
Mn-Zr	-2.8371	-1.417	0.727	0.488
Mo-Mo	0.3166	-1.131	0.388	1.443
Mo-Ru	0.3197	-1.232	0.562	0.656
Nb-Mo	-0.1454	-0.671	0.219	1.219
Nb-Zn	-0.5799	-1.467	-0.003	1.297
Ni-Rh	-1.5834	-0.891	1.015	-0.779
Pd-Rh	-0.7361	-1.003	1.026	-0.333
Ti-Mo	-0.8656	-2.015	-0.838	0.118
Ti-Nb	-1.2029	-1.616	-1.577	2.085
V-Cr	-1.5199	-1.676	-0.373	1.216
V-Ru	-1.0207	-1.022	0.578	0.212
Zr-Cd	-2.6825	-	-	-

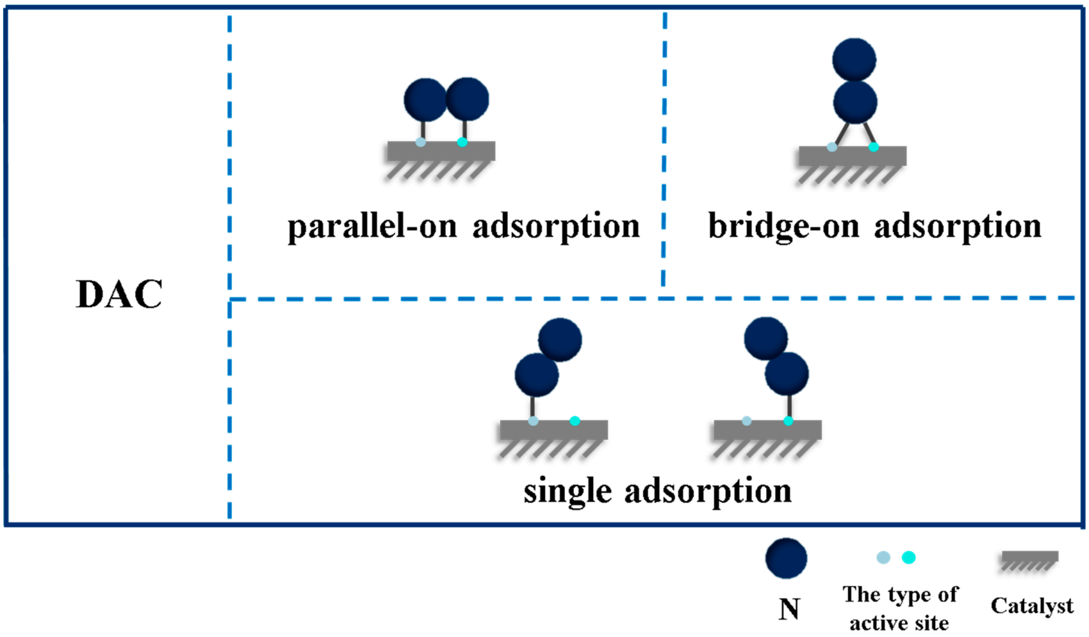

Figure S1. Schematic diagram of common adsorption structures of NRR

The ML models employed in this work were trained on DFT computational data for diatomic nitrogen-doped graphene catalysts, specifically focusing on binding energies in nitrogen reduction reactions, adsorption energies of key intermediates, first-step hydrogenation energy changes, and final-step hydrogenation energy changes. The dataset encompasses relevant structural characteristics including electronic properties such as metallic electronegativity, electron affinity, first ionisation energy, and d-electron count. Features were normalised and redundant or weakly correlated attributes removed to retain those most pertinent to adsorption energy prediction.

The dataset was partitioned into training and test sets at an 80:20 ratio. Within the training set, model selection and hyperparameter optimisation underwent four-fold cross-validation. The cross-validation process ensured robust model performance while minimising overfitting. Model performance was evaluated using multiple metrics, including root mean square error (RMSE) and coefficient of determination (R^2). Supplementary figures provide comparisons between predicted and calculated values for both training and test sets, demonstrating the predictive accuracy of the ML model. As illustrated in Figure 5(a-d), the RFR prediction results are generally consistent with the DFT calculation results. The RMSE is less than 0.18, and the R^2 is greater than 0.9, which provides a solid foundation for subsequent research.These details ensure the reproducibility and reliability of the ML-assisted DFT workflow.

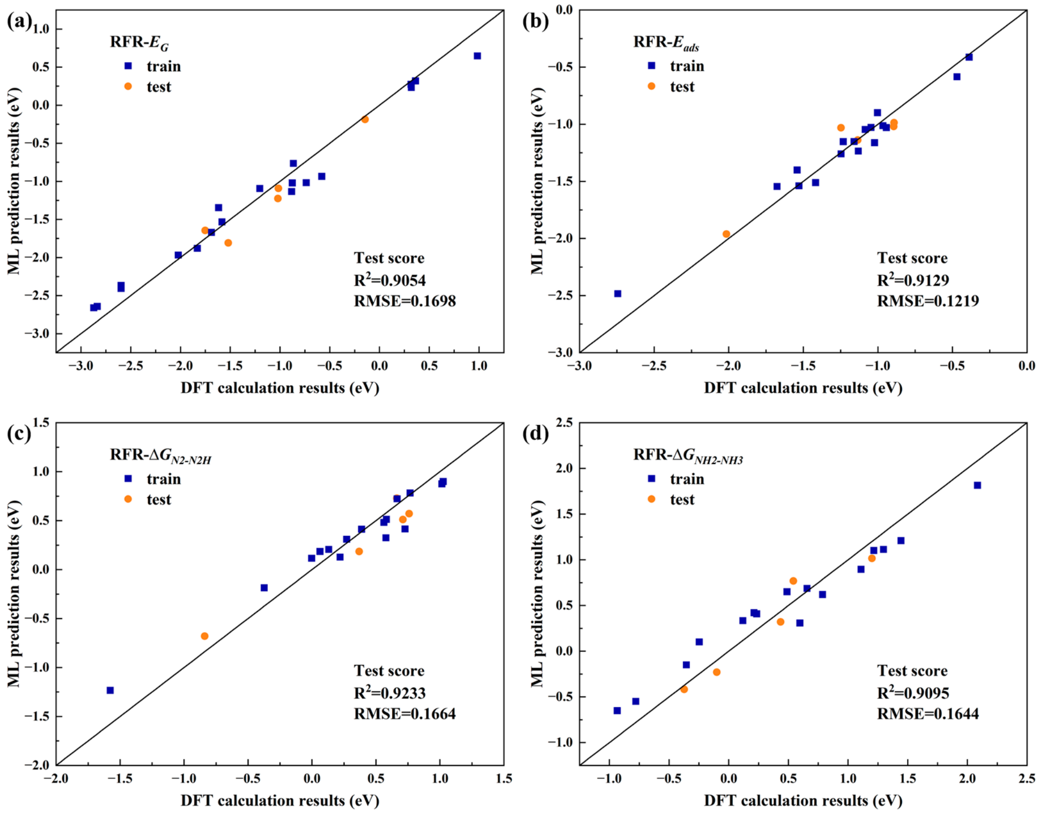

Figure S2. Comparison between DFT calculation results and RFR predictions for (a) $\boldsymbol{E_G}$ (b) $\boldsymbol{E_{ads}}$ (c) $\Delta\mathbf{G}_{\mathbf{N_2-N_2H}}$ and (d) $\Delta\mathbf{G}_{\mathbf{NH_2-NH_3}}$
